# Vision-Based Estimation of Force Balance of Near-Suspended Melt Pool for Drooping and Collapsing Prediction

**DOI:** 10.3390/s24113270

**Published:** 2024-05-21

**Authors:** Longxi Luo, Enze Qian, Tao Lu, Jingren Pan, Minghao Liu, Changmeng Liu, Yueling Guo, Luzheng Bi

**Affiliations:** School of Mechanical Engineering, Beijing Institute of Technology, Beijing 100080, China; longxiluo@bit.edu.cn (L.L.); 3220230313@bit.edu.cn (E.Q.);

**Keywords:** wire-arc additive manufacturing, computer vision, image segmentation, near-suspended melt pool, perception and monitoring

## Abstract

Wire-arc additive manufacturing (WAAM) is favored by the industry for its high material utilization rate and low cost. However, wire-arc additive manufacturing of lattice structures faces problems with forming accuracy such as broken rod and surface morphology defects, which cannot meet the industrial demand. This article innovatively combines the melt pool stress theory with visual perception algorithms to visually study the force balance of the near-suspended melt pool to predict the state of the melt pool. First, the method for melt pool segmentation was studied. The results show that the optimized U-net achieved high accuracy in melt pool segmentation tasks, with accuracies of 98.18%, MIOU 96.64%, and Recall 98.34%. In addition, a method for estimating melt pool force balance and predicting normal, sagging, and collapsing states of the melt pool is proposed. By combining experimental testing with computer vision technology, an analysis of the force balance of the melt pool during the inclined rod forming process was conducted, showing a prediction rate as high as 90% for the testing set. By using this method, monitoring and predicting the state of the melt pool is achieved, preemptively avoiding issues of broken rods during the printing process. This approach can effectively assist in adjusting process parameters and improving welding quality. The application of this method will further promote the development of intelligent unmanned WAAM and provide some references for the development of artificial intelligence monitoring systems in the manufacturing field.

## 1. Introduction

Additive manufacturing technology is a new manufacturing technology based on the principle of layered dispersion and layered stacking, which realizes material molding through the “bottom-up” process [1,2,3]. This technology can quickly and accurately manufacture objects with complex shapes [4]. In addition, it has the advantages of simple structure, low technical cost, and high manufacturing efficiency [5]. Additive manufacturing technology has been widely used in automobile, aerospace, medical, and other industrial fields [6], and therefore has the potential to solve the bottleneck problem of rapid manufacturing of complex structural parts.

Based on the classification of heat sources, metal additive manufacturing can be divided into high-energy beam additive manufacturing, electron-beam additive manufacturing technology, and wire-arc additive manufacturing technology (such as wire arc and plasma arc). Among them, wire-arc additive manufacturing, also known as wire-feeding additive manufacturing, is generally based on the process of continuous melting and accumulation of metal wires under the action of the welding heat source [7]. There are three kinds of WAAM methods: gas metal WAAM (GMA-AM), plasma WAAM (PA-AM), and gas tungsten WAAM (GTA-AM). In contrast to traditional subtractive processing technology, the wire-arc additive manufacturing technology significantly reduces processing steps and enables the fabrication of intricate spatial structures. Traditional subtractive manufacturing methods such as investment casting [8], plastic processing, and extrusion combined with electrical discharge machining, have stringent material requirements that constrain the design flexibility for lattice structures. They also necessitate mold design, are cost-intensive, and involve cumbersome processes, making them unsuitable for large-scale lattice complex structures. When compared with the high-energy beam additive manufacturing technology, the wire-arc additive manufacturing technology stands out as a cost-effective and unsupported solution for spatial structure fabrication. The high-energy beam additive manufacturing necessitates spreading metal powder on a pre-printed layer and precisely controlling the laser beam emitted by a high-power density laser through optical lenses to selectively melt the powder. However, the equipment and powder required in this technology are prohibitively expensive, its deposition rate is low, and the equipment’s spatial constraints limit its applicability to small-sized lattice structures [9,10]. In general, the wire-arc additive manufacturing technology has an immense application prospect and a pivotal role in the manufacturing and development of metal lattice structures.

Over the last five years, the production of lattice structures via WAAM has emerged as a burgeoning research area. At present, the majority of related studies leverage industrial robots as platforms to fabricate lattice structures utilizing inert gas-shielded WAAM technology, specifically WAAM-GMAW [11]. Li et al. [12] used the WAAM-GMAW method to manufacture aluminum alloy multi-angle inclined rods and large multi-layer lattice structures through multi-axis welding robots. Wu et al. [13] realized the fabrication of an aluminum alloy pyramid lattice based on WAAM-GWAM. In this study, a workbench with multiple degrees of freedom and a welding torch fixed in the Z direction were used.

The additive manufacturing research group in the Beijing Institute of Technology has developed the hot filament pulsed arc, unsupported arc, and multi-arc parallel additive manufacturing methods to form an efficient additive manufacturing technology of unsupported lattice sandwich structure. Mao et al. [14] developed the pulsed-arc WAAM method, which uses pulse current to make the melt pool melt at the peak time and solidify at the base time. The melt pool cools during the base arc, and a rod is continuously formed before the next rod is prepared. This approach significantly reduces time, eliminating the need for arc pausing and welding torch relocation. Jing et al. [15] used a pulse arc to control the flow of melt pool in WAAM to realize the non-supported manufacturing of rods with various inclination angles without the need to adjust the welding torch or workbench. In addition, the concept of efficient additive manufacturing based on multi-heat sources is developed, and the efficient manufacturing method of multi-arc large lattice structure is developed, which greatly improves the manufacturing efficiency of lattice structure. Despite the fact that the research team has made a preliminary study on the manufacturing method, forming the mechanism and basic manufacturing equipment design of WAAM for lattice sandwich structure, it is difficult to avoid the forming quality problems in the process, such as broken rods, surface morphology defects of rods (bulges, fused unmelted wires, roughness, and burrs), shape and position errors of rods (uneven change or error in diameter, uneven change or error in inclination angle, rod twisting, and rod bending), and rods in the same lattice cell being too far apart. Notably, rod breakage is unacceptable, which may be caused by the collapse of the melt pool due to unbalanced force or arc instability resulting from an inappropriate tungsten electrode–melt pool distance. The complexity and instability of the nearly suspended melt pool in the process of lattice structure manufacturing make it more likely to cause rod breakage or surface morphology defects. Therefore, to mitigate the risk of rod breakage and morphological defects, it is imperative to continuously monitor the distinct characteristics of the near-suspended melt pool.

At present, the control of forming accuracy mainly depends on the operator’s eye observation and manual control, but the efficiency of manual control is limited and depends on experience and therefore cannot meet the requirements of autonomy, efficiency, and high precision for WAAM of large-scale lattice structures. Therefore, the lack of self-control ability and forming accuracy has become the key technical bottleneck restricting the advancement and scale application of the WAAM of lattice structures. It is urgent to develop intelligent and unmanned WAAM for lattice structures to improve their production quality.

In recent years, computer vision and deep learning have been widely used in many fields, mainly for visual perception in robot, unmanned aerial vehicle, rescue search, and other fields, which also extended to additive manufacturing and welding related fields for monitoring the visual characteristics of the melt pool. Le et al. [16] developed a set of in situ monitoring systems for melt pool detection based on vision, which realized the monitoring of melt pool boundaries. He et al. [17] used a passive vision sensor to obtain the visual appearance of the WAAM melt pool, and proposed a quantitative prediction method of deposit drift. This method uses deposit data to build a model and predict the drift and can be used to monitor the deposit drift in real-time. Xia et al. [18] applied deep learning visual monitoring to WAAM, studied and compared the melt pool classification performance of various convolutional neural models, and collected different abnormal melt pool images for training. The results show that the CNN model can effectively classify different types of melt pool images. Zhang et al. [19] established a WAAM melt pool early warning system. This system not only uses a convolutional neural network to construct a melt pool image classification model, but also uses a Grad-CAM algorithm to visually analyze the model, which can classify melt pool images into splash, collapse, short overlapping distance, long overlapping distance, normal overlapping, and normal stacking categories. Shin et al. [20] studied an adaptive anomaly detection method to solve the problem of scarce experimental data of high-cost materials. This method uses attribute cascade transfer learning to transfer enough datasets of low-cost materials to high-cost materials, so as to accurately detect spherical defects of high-cost materials in melt pool images.

Overall, there have been plenty of studies on improving WAAM through robotics, process design, and monitoring. However, the research on the force balance of the melt pool and collapsing prediction for visual monitoring of the WAAM of lattice structures is scarce. Based on this, this paper conducts a vision-based study to monitor the melt pool forming process. This study captures the WAAM process via a camera; processes and analyzes the video stream by developing computer vison methods, including segmenting the melt pool area, estimating the upper contact angle and the lower contact angle of the melt pool, so as to evaluate the force balance of the melt pool; and predicts the drooping and collapsing state of the melt pool. This study provides an explainable melt-pool-state prediction method that can guide the fine-tuning of process parameters for maintaining the stability of the melt pool forming and accumulation processes, thus improving the forming quality of WAAM.

## 2. Methods

### 2.1. Principle

Distinct from the conventional layer-by-layer stacking support approach, the WAAM methodology employs an arc as the heat source. It solidifies droplets in a pulsed, layer-by-layer fashion, continuously fashioning solidified metal rods in a fixed direction as the welding torch moves. Consequently, employing WAAM significantly enhances material utilization efficiency. In the experiment, the state of the melt pool has a profound influence on the formation of a nearly suspended rod. Hence, the most important problem is to predict the state of the melt pool (normal, drooping, or collapsing), so as to realize the manufacture of nearly suspended inclined rods by adjusting process parameters.

**Figure 1:**
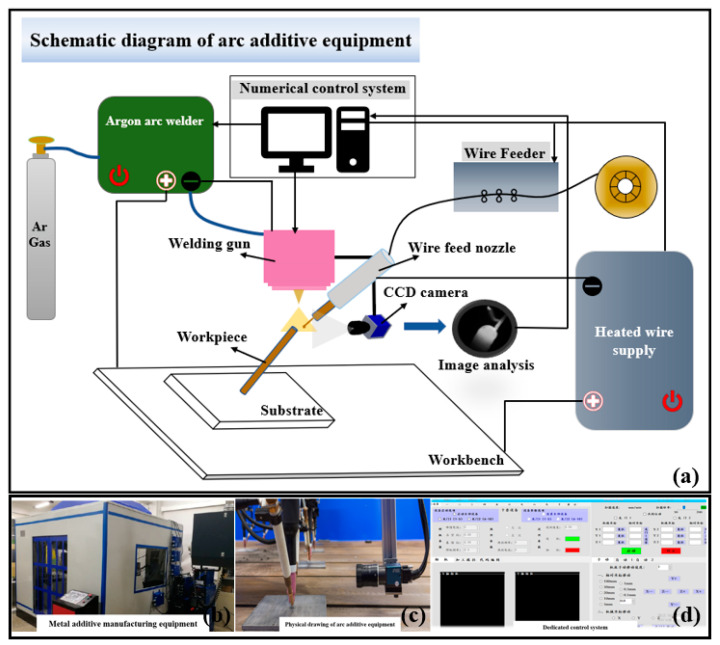
Schematic plot of the WAAM process with visual monitoring system: (**a**) schematic diagram of printing, monitoring, and control hardware; (**b**) overview of WAAM equipment; (**c**) image printing head and video camera installation; and (**d**) control system UI.

**Figure 2:**
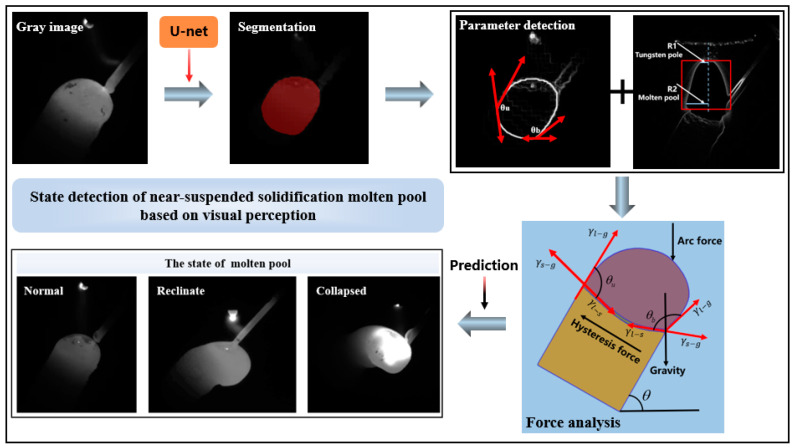
Method of force balance estimation of nearly suspended solidifying melt pool based on visual perception.

Figure 1 showcases the visual monitoring and control system seamlessly integrated into the large-scale WAAM equipment. Meanwhile, Figure 2 highlights the core principles of the vision-based force balance estimation method of the near-suspended melt pool proposed in this study. The melt pool changes during the manufacturing process of lattice rods are captured with a video camera in the WAAM experiment. Utilizing deep learning, we detect the characteristics of the melt pool, and employ a computer vision algorithm to determine the upper and lower contact angles for force balance analysis. This enables us to predict the state of the melt pool (normal or otherwise) and promptly feed the results back to the controller. By adjusting the process parameters accordingly, we can enhance the manufacturing quality of near-suspended lattice rods.

### 2.2. Image Segmentation of the Melt Pool

#### 2.2.1. Image Processing

To effectively apply supervised deep learning for melt pool condition monitoring, it is imperative to curate a substantial amount of annotated training data. Inaccurate labels in the training dataset will derail the trained deep learning model, resulting in the model being unable to accurately measure the contact angle. In addition, given the scarcity of existing melt pool image datasets, most of which are non-public, we have compiled a comprehensive set of melt pool image data for the purpose of training the deep learning model.

Utilizing the aforementioned experimental equipment, videos capturing the state changes in the melt pool during the WAAM process were gathered. The video duration was about 20 min, encompassing wire feeding, melting, deposition, and solidification. Image-based deep learning actually concerns data analysis of every pixel of the image, and a video is composed of multiple consecutive frames of images. This study intercepts 20 frames every second of the video, extracts and stores one frame every 10 frames, and finally acquires 1500 images with a 960 × 544 resolution. However, due to factors, such as camera exposure and early deposition, that can impact video quality, it was crucial to filter the captured melt pool images. After eliminating images with issues like blurred fields of view and indistinct deposited layers, we retained a total of 600 effective melt pool images for further analysis.

#### 2.2.2. Dataset

Due to the indistinct boundary between the melt pool layer and the deposited layer, this study employed manual annotation of the melt pool contour, leveraging the theoretical definition of the melt pool and the expertise of professional researchers. Labelme, an image annotation tool based on Python and Qt with a graphical interface, is used for annotation. After manually annotating 600 melt pool images, the same number of files with a JSON format are obtained, and 600 images with PNG format are obtained via batch processing and format conversion, which is convenient for neural network training. Ultimately, the complete dataset is divided according to the ratio of 8:1:1 to obtain training, validation, and testing sets.

#### 2.2.3. Structure of the Network

Due to its numerous advantages, the segmentation of the melt pool is accomplished using a segmentation model based on the foundations of U-net [21] and ResNet50 [22]. In 2014, Long et al. [23] proposed a full convolution network (FCN), which realized end-to-end semantic segmentation at the pixel level based on a CNN, allowing the CNN to make dense pixel predictions without a complete connection layer. Subsequently, Ronneberger et al. [21] proposed the U-net network based on an FCN and applied it to medical image segmentation. As a typical encoder–decoder network, the U-net architecture not only ensures consistency in resolution between the output and input layers but also integrates low-dimensional and high-dimensional features of the FCN through its symmetrical U-shaped design. In the realm of semantic segmentation networks, the up-sampling methods of nearest-neighbor interpolation [24] and bilinear interpolation [25] are usually adopted. As an example, Lin et al. [26] proposed a top-down network structure with lateral connections, creating feature maps that encapsulate advanced semantic information of varying sizes. Specifically, they employed the nearest-neighbor interpolation method, which maximizes the retention of semantic information during the up-sampling process, benefiting classification. This method enables seamless integration with feature maps rich in high-resolution spatial information during the bottom-up process. The resulting fusion layer then amalgamates various network branches. By merging the high-resolution, low-level features of location information with low-resolution, high-level features of semantic information learned via the convolutional neural networks, a more comprehensive array of image features can be extracted.

**Figure 3:**
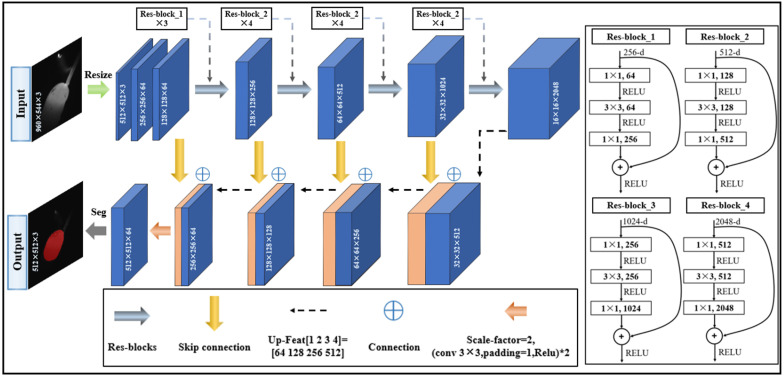
Detailed structure of the proposed melt pool segmentation model.

The structure of the proposed melt pool segmentation model is shown in Figure 3. The model uses an approximately symmetrical encoder–decoder structure composed of 9 units, including 4 down-sampling units, 4 up-sampling units, and 1 fusion unit. The original image size is 960 h × 544 w, the input images are resized to 512 h × 512 w, and the output image size is 512 h × 512 w. The upper part of the melt pool segmentation model consists of five units, and its backbone structure adopts ResNet50, including four residual blocks which are used to extract high-dimensional features by gradually reducing the spatial dimensions of the input image. The fifth unit is the end of down-sampling ahead of up-sampling. The lower part of the network has four units, which gradually restore the down-sampled high-dimensional features to their original resolution. The dimension changes in the characteristic layer of each cell are shown in Table 1 and Table 2.

#### 2.2.4. Model Training and Testing

The super-parameters for online learning are set according to experience and related research [27]. The Stochastic Gradient Descent (SGD) [28] method and the Adaptive Moment Estimation (Adam) [29] optimizer are used to train the network, initiating the learning rate at 0.0001. In this study, a small batch learning method is adopted, setting the batch size to 2, thus processing two samples (in the form of three-dimensional adjacency matrices) in each iteration. Furthermore, we have conducted a total of 300 rounds of training, saved the network parameters, and updated the optimal network parameters every 5 rounds. The proposed model is implemented in Pytorch and trained on a computer with Intel (R) Core (TM) i7-10750h CPU @ 2.60 GHz, with 16 GB RAM, and a NVIDIA GeForce GTX 3080 GPU.

### 2.3. Force Balance Estimation for Melt Pool

Due to the influence of surface tension, gravity, arc force, and viscous force on the fluid within the melt pool, it is necessary to accurately obtain all composition forces in order to analyze its force balance. Among them, the upper and lower contact angles for calculating melt pool viscous force, and the arc radius of peak arc for calculating arc force are the most difficult parameters to measure. Based on the segmentation of the melt pool, the upper and lower contact angles and arc radius can be obtained via computer vision methods.

#### 2.3.1. Contact Angle Estimation

**Figure 4:**
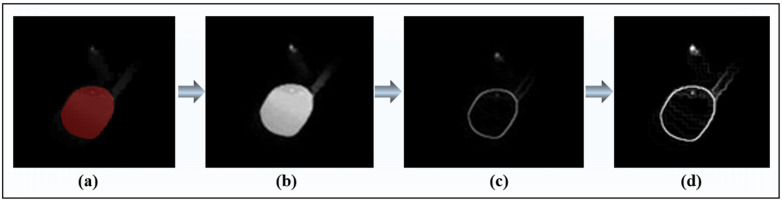
Edge profile extraction of melt pool: (**a**) melt pool segmentation image; (**b**) binarization of melt pool segmentation image; (**c**) extracting edge profile of melt pool based on Sobel operator; and (**d**) image enhancement.

As an image is composed of a plurality of pixels and the packing density of pixels is high, in order to find the required angle accurately and quickly, the segmented melt pool image needs to be preprocessed and enhanced. The melt pool image is extracted based on semantic segmentation, as shown in Figure 4a, but the estimation of the contact angle is mainly based on the edge profile of the melt pool, so the edge profile of the melt pool is extracted by applying a binarization algorithm and a Sobel operator, as shown in Figure 4b,c. Furthermore, to enhance the visibility of crucial features, this study enhanced the edge contour of the melt pool, facilitating more effective feature extraction, as illustrated in Figure 4d.

**Figure 5:**
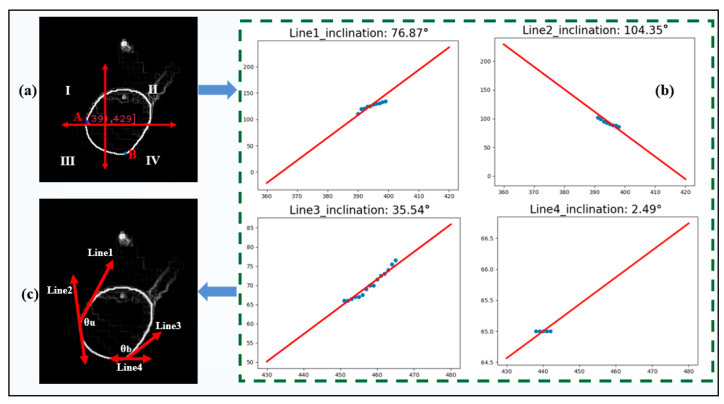
Estimating the contact angle of the melt pool: (**a**) edge region division in the melt pool image; (**b**) tangent line fitting using the Ransac algorithm; and (**c**) schematic plot of the upper and lower contact angles of the melt pool.

The edge contour of the melt pool is extracted with the above method. Then, the critical turning points (A and B) of the upper and lower contact angles of the melt pool are identified by finding the leftmost point and the bottom point. The coordinates of all pixels along the edge profile of the melt pool are obtained and divided into four regions: I, II, III, and IV, as shown in Figure 5a, making it easy to obtain the upper and lower contact angles of the melt pool. Then, based on the Ransac algorithm [30], all the pixel points in the edge contour areas of three melt pools, I, III, and IV, are traversed, respectively, and tangents of both turning points A and B are obtained, respectively, as shown in Figure 5b. Based on the slopes and angles of the tangent lines, the upper contact angle *θ_u_* and the lower contact angle *θ_b_* of the melt pool are obtained, as shown in Figure 5c.

#### 2.3.2. Arc Radius Detection

**Figure 6:**
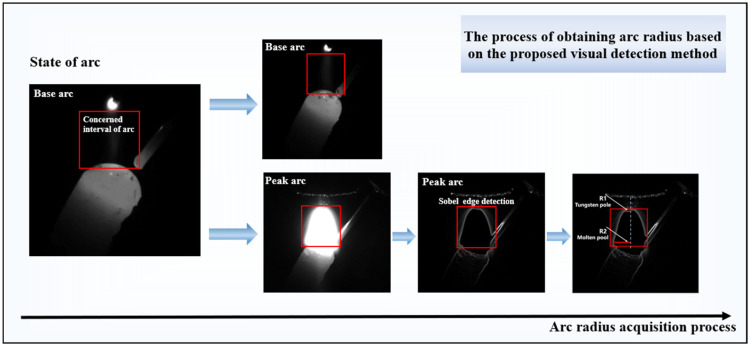
The arc radii are obtained based on the proposed visual detection method.

The detection of the arc radius is based on the arc image recorded via a CCD camera during the peak arc duration. Specifically, R1 represents the arc radius near the tungsten electrode, while R2 denotes the arc radius at the melt pool, as illustrated in Figure 6. When the current is at the base value, the arc image is shown as a base arc image, and when it is at the peak value, the arc image is shown as a peak arc image. Distinguishing between these two images is straightforward, achieved by calculating the average intensity value within the region of interest, highlighted by the red rectangle in Figure 6. Utilizing the peak arc image as input data, we employ the Sobel operator to precisely extract the arc’s edge contour. Finally, the pixel radii of *R*_1_ and *R*_2_ are estimated based on the extracted edge contour.

In order to convert these pixel distances into actual distances, we adopted Formula (1). In this conversion process, a crucial parameter is the actual size of each pixel, referred to as the scale factor. The scale factor can be obtained as the ratio of the physical size of a known object over its pixel size in the image. The scale factor of this study was calculated as 0.294 mm/pixel. Finally, based on the calculation of Formula (1), we can obtain the specific values of arc radius *R*_1_ at the tungsten tip and arc radius *R*_2_ at the top of the melt pool. This detection process not only ensures the accuracy of measurement, but also provides invaluable data support for our subsequent analytical and research endeavors.

Actual size = pixel size × scale factor(1)

#### 2.3.3. Quantitative Definition of Drooping and Collapsing Melt Pool States

**Figure 7:**
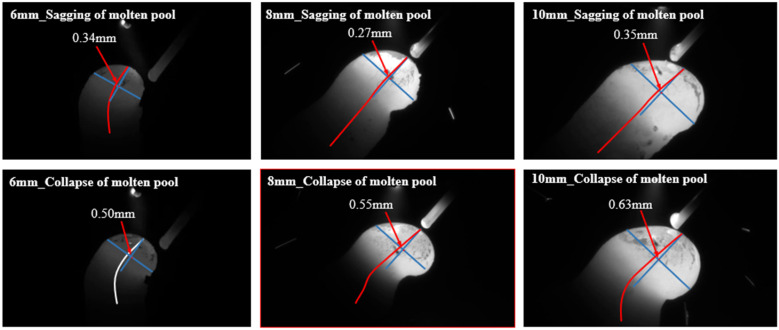
The distinction between drooping and collapsing state of melt pool.

At present, the research on predicting melt pool state (drooping and collapsing) is still insufficient, and there is a lack of a comprehensive and systematic quantification and definition to distinguish normal, drooping, and collapsing melt pool states. In view of this, this study proposes a new method to define and quantitatively distinguish states based on the deviation rate of the center of gravity of the melt pool. Through experimental analysis, we observed that changing the duty ratio of peak arc will lead to a change in the melt pool state, but the boundary between the drooping and collapsing states is still not clear enough. However, compared with the normal state, a common feature of the drooping and collapsing states is that the center of gravity of the melt pool will deviate from the axis of the rod. In addition, we also find that there are differences in the offset distance of the center of gravity between the two abnormal melt pool states. By observing image data from experiments with different non-supported rods with 6 mm, 8 mm, and 10 mm diameters, we set a range of offset rates to define the two different states, as shown in Figure 7. Specifically, if the ratio of the deviation distance of the melt pool’s center of gravity to the rod’s axis falls within the range of 5% to 12%, we consider the melt pool to be in a drooping state, indicating a downward trend. However, timely adjustments to the duty ratio can restore the bar to its normal manufacturing condition. Conversely, when this ratio surpasses 12%, we deem the melt pool to be in a collapsed state. If the duty ratio at that moment remains unchanged or even increases, the phenomenon of rod breakage will occur, and the manufacturing will not be able continue. This method offers us a robust instrument for precisely identifying and analyzing the state of the melt pool.

#### 2.3.4. Force Analysis

Based on the above visual-based methods and known conditions, the viscous force, gravity force, and arc force are calculated, respectively.

The viscous force [31] *F_h_* is calculated as follows:

(2) $$F_{h}=\pi D\gamma \left(\left|\cos\theta_{b}\right|-\left|\cos\theta_{u}\right|\right)$$

where D is the rod diameter, and *θ_u_* and *θ_b_* are the upper contact angle and lower contact angle of the melt pool, respectively, and their values are already obtained.

For surface tension γ, McNallan and Debroy [32] provided the calculation method in the Fe-Cr-Ni-S system:

(3) $$\gamma =\gamma_{0}-4.3\times 10^{-4}\left(T-T_{0}\right)-RT\Gamma_{s}\ln(1+K_{s}a_{s}e^{\left(-\frac{\Delta H^{o}}{RT}\right)})N/m$$

where *γ*_0_ is the surface tension of pure metal at reference temperature T_0_, T is the current temperature of the workpiece, and 304 L and 316 L are about 1673 K; when Γ_S_ is saturated and there is surface excess. Sahoo et al. [33] provide the values of *γ*_0_ and Γ_S_ as 1.943 N/m and 1.3 × 10^−8^ Kg·Mole/m^2^, respectively, at the temperature of 1809 K; K_s_ and ΔH^o^ represent the entropy and enthalpy factors in the sulfur adsorption reaction, and their values are 0.00318 and −1.662 × 10^8^ J/(K·Kg·Mole), respectively. R is a gas constant with a value of 8.314 × 10^3^ J/(K·Kg·Mole); a_s_ is the activity of sulfur in solution, and its calculation formula is as follows:

(4) $$a_{s}=\left[pctS\right]\cdot 10^{\left\{\left[-\frac{94.2}{T}+0.0396\right]*\left[pctCr\right]\right\}}$$

where *pctS* is the sulfur content in the melt pool and *pctCr* is the chromium content in the melt pool. According to the wire information provided by the manufacturer, the values of *pctS* and *pctCr* in 316 L stainless steel are 0.013 and 19, respectively.

The arc force *F*_arc_ is calculated as follows [34]:

(5) $$F_{arc}=\frac{\mu_{0}}{8\pi }I^{2}\left(1+2\ln\left(\frac{R_{2}}{R_{1}}\right)\right)$$

where *μ*_0_ is the vacuum permeability with a value of 4π × 10^−7^ N/A^2^, *I* is the arc current, *R*_1_ is the arc radius of the tungsten electrode, and *R*_2_ is the arc radius at the melt pool.

In order to predict the state of the melt pool, we need to analyze the balance of the acting forces calculated above. It is very important to ensure that the viscous force exceeds the sum of the gravity force and arc force in the radial direction of the rod in order to avoid collapsing of the melt pool when preparing the rod with a positive inclination angle. The following formula needs to be satisfied to maintain a balanced condition.

(6) $$F_{h}\geq \left(F_{g}+F_{arc}\right)\cos\theta$$

where θ is the inclination angle of the printing rod, and, in this paper, the inclination angle without a support rod is 45°.

If the above formula is not satisfied and the value on the right side of the above formula is obviously larger than that on the left side, then it is predicted that the melt pool is collapsing. If the values on both sides of the formula are similar (within a 5% difference), then the melt pool is predicted to be drooping. Otherwise, the melt pool is determined to be normal and stable. At the same time, based on this formula, we can also effectively measure the maximum allowed mass of the melt pool.

(7) $$M\leq \frac{1}{g}\left(\frac{F_{h}}{\cos\theta }-F_{arc}\right)$$

The above Formulas (2)–(5) are supported and confirmed by relevant research and can be referred to relevant literature [31,32,33,34], so there is no need to conduct numerical analysis through design experiments.

## 3. Experiment

### 3.1. Experimental Setup

The principle of WAAM involved in this paper is based on tungsten inert gas-shielded welding technology, which has the advantages of low spatter, low cost, and flexible control. The experimental system can be divided into three parts: the WAAM system, the numerical control system (CNC), and the video camera monitoring system. The experimental hardware consists of an argon-arc welding machine, a hot wire power supply, a wire feeder, a numerical control system, argon gas, and a machine tool, as shown in Figure 1.

The experimental system of WAAM used in this research uses a ten-head array WAAM equipment developed by the authors’ group. The argon arc welder, wire feeder, and hot wire power supply adopt the WSM-400R(HW), ZSS-G(T), and HW-200 models of Shandong Aotai Electric Co., Ltd. (Tianjin, China), respectively. These devices have data monitoring function which can monitor the welding machine current, voltage, wire feeding speed, and hot wire current. The cathode of the argon arc welding machine is seamlessly integrated with the tungsten electrode on the welding gun, while the anode is securely connected to the workbench. When the argon arc welding machine is turned on, a high-energy arc can be generated between the workpiece on the workbench and the tungsten electrode. The negative pole of the hot wire power supply connects to the wire material via a conductive nozzle, and the positive pole is connected with the workbench. When the wire comes into contact with the workpiece on the workbench, the hot wire current will form a loop. Due to the thin wire and high resistance between the contact tip and the workpiece, significant resistance heat is generated to effectively preheat the wire. The function of the wire feeder is to feed the wire into the melt pool at a set speed. The numerical control system is used to control the movement of machine tools, parameters of the welding machine and hot wire machine, wire feeding speed, etc. Argon serves as a crucial protective agent for the melt pool, shielding it from oxygen and preventing oxidation, while also facilitating arc ignition. The welding gun is fixed on the spindle of the machine tool, and the machine tool drives the welding gun to move.

The Computer Numerical Control system, which is independently developed by the authors’ group, can control the parameters and start–stop state of machine tool movement, the welding machine, the hot wire power supply and the wire feeder. The video camera system adopts the second-generation model MER2-041-302GM/C from ShuiXing and is equipped with a fixed-focus lens, model HN-P-5028-10M-C2/3. The frame rate of the camera is 30 fps, and the pixel size is 6.9 μm × 6.9 μm. The camera, wire feeder, and welding gun are fixed on the machine tool. As the welding gun moves, the camera follows, capturing real-time visuals of the evolving melt pool and rod during the additive manufacturing process. The images taken by the video camera system are finally transmitted to the computer for detailed analysis and processing.

### 3.2. Material Parameter

The material required for the experiment is 316 L austenitic stainless steel and the wire diameter is 1.2 mm. The chemical composition of 316 L stainless steel is shown in Table 3, and the material composition is provided by Beijing Jinwei Welding Material Co., Ltd. with a density of 7.89 g/cm^3^. The material was selected because austenitic stainless steel has good weldability, corrosion resistance, and high-strength mechanical properties.

### 3.3. Experimental Design

To validate the effectiveness of the force balance estimation for the near-suspended solidifying melt pool, based on visual perception techniques proposed in this study, we have devised a comprehensive experimental scheme for rigorous verification. The inclined angle of the near-suspended rod designed in this study is 45°, and three groups of control experiments with the same manufacturing process are set up. The diameters of the three groups of rods are 6 mm, 8 mm, and 10 mm, respectively. For each group of experiments, the total time is 2 min, and each group is divided into four cases of control experiments. Take the experimental control group with a rod diameter of 6 mm as an example. In the first case of control experiments, the manufacturing process of the first group of experiments is normal manufacturing in the first 30 s, the melt pool sags during this period by changing the current or duty ratio in 30 to 60 s, returns to the normal manufacturing process during 60 to 90 s, and tends to collapse by changing the current or duty ratio in 90 to 120 s. The manufacturing process of the second case of experiments is somewhat different from that of the first case; that is, the time period of the drooping state and the collapsing state of the melt pool are exchanged. In the third case of control experiments, during the manufacturing process, the first 45 s is normal manufacturing, the melt pool sags in the duration from 45 s to 75 s by changing the current or duty ratio and returns to the normal manufacturing process from 75 s to 90 s, and the melt pool collapses during this period from 90 s to 120 s by changing the current or duty ratio. The fourth case is similar to the third case except that the durations for rod drooping and collapsing are exchanged. The above practice is designed to improve the randomness of the experiment and verify the robustness of the model. The detailed experimental scheme is shown in Table 4.

## 4. Results and Analysis

### 4.1. Evaluation of Network

**Figure 8:**
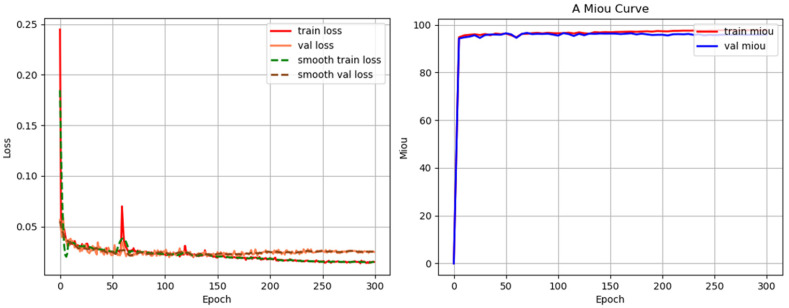
Training/validation loss curve and MIOU value of the segmentation model.

A total of 600 images were collected and split into training, validation, and testing datasets with a ratio of 8:1:1. The segmentation performance has been rigorously evaluated. Figure 8 exhibits the training/validation loss curve of the improved model based on U-net and the variation curve of MIOU value of the training set and validation set. After 15 epochs, all models gradually converge in terms of training and validation loss and MIOU index. Notably, after 200 epochs of training, the model achieves a training loss of less than 0.01 and a validation loss of less than 0.02.

**Figure 9:**
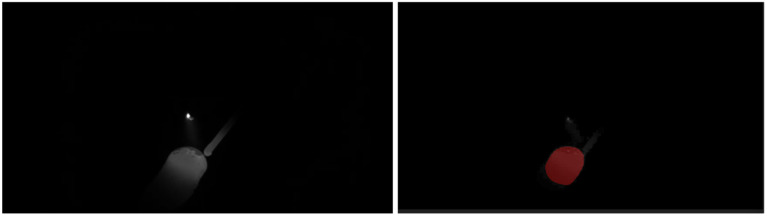
Example of the segmentation test results based on melt pool segmentation model.

**Figure 10:**
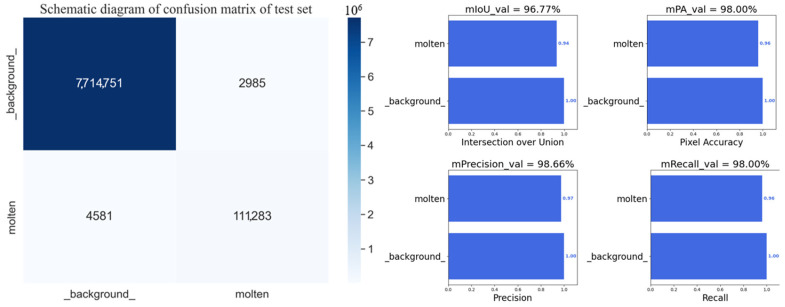
Confusion matrix of melt pool segmentation for the validation set.

**Figure 11:**
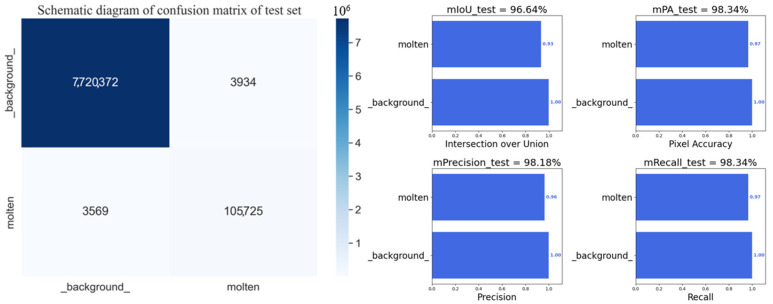
Confusion matrix of melt pool segmentation for the testing set.

Figure 9 presents an example of the results of melt pool segmentation using the best-trained network parameters with the best training effect. The melt pool image was randomly chosen for illustration, and the segmented area is highlighted in red. Figure 10 provides a detailed breakdown of the model’s performance on the validation set, revealing an impressive average accuracy of 98.66%, a Mean Intersection over Union (MIOU) of 96.77%, and an average recall of 98.00%. In addition, in order to further verify the robustness of the model, the model was evaluated on the testing dataset, and the precision and confusion matrix values of the testing set are presented in Figure 11. In the testing set, the model still achieved stable and high accuracy, with an average accuracy of 98.18%, a MIOU of 96.64%, and an average recall of 98.34%. All indicators demonstrate that the model has good ability to segment melt pools, which may be a challenging task for human experts.

### 4.2. Interpretability

**Figure 12:**
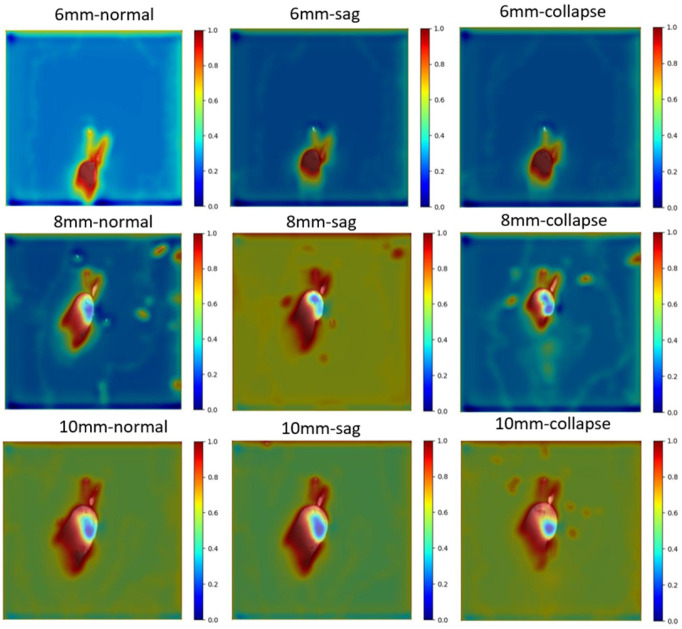
Heat map based on Grad-CAM.

To facilitate a deeper understanding of our design, we present some visual results. Given that heat maps effectively illustrate a model’s focus on input data, for the purpose of target segmentation in this study, our model is capable of generating a heat map that highlights the regions of interest within an image. Drawing inspiration from this concept, we integrated Grad-CAM into our network and segmented the melt pool of non-supportted rods using the melt pool segmentation model (as shown in Figure 12). The resulting visualizations demonstrate that the network indeed attends to the precise areas of interest, thus validating the interpretability of our model’s decision-making process.

### 4.3. Experimental Verification of Force Estimation and Drooping and Collapsing Prediction Methods

**Figure 13:**
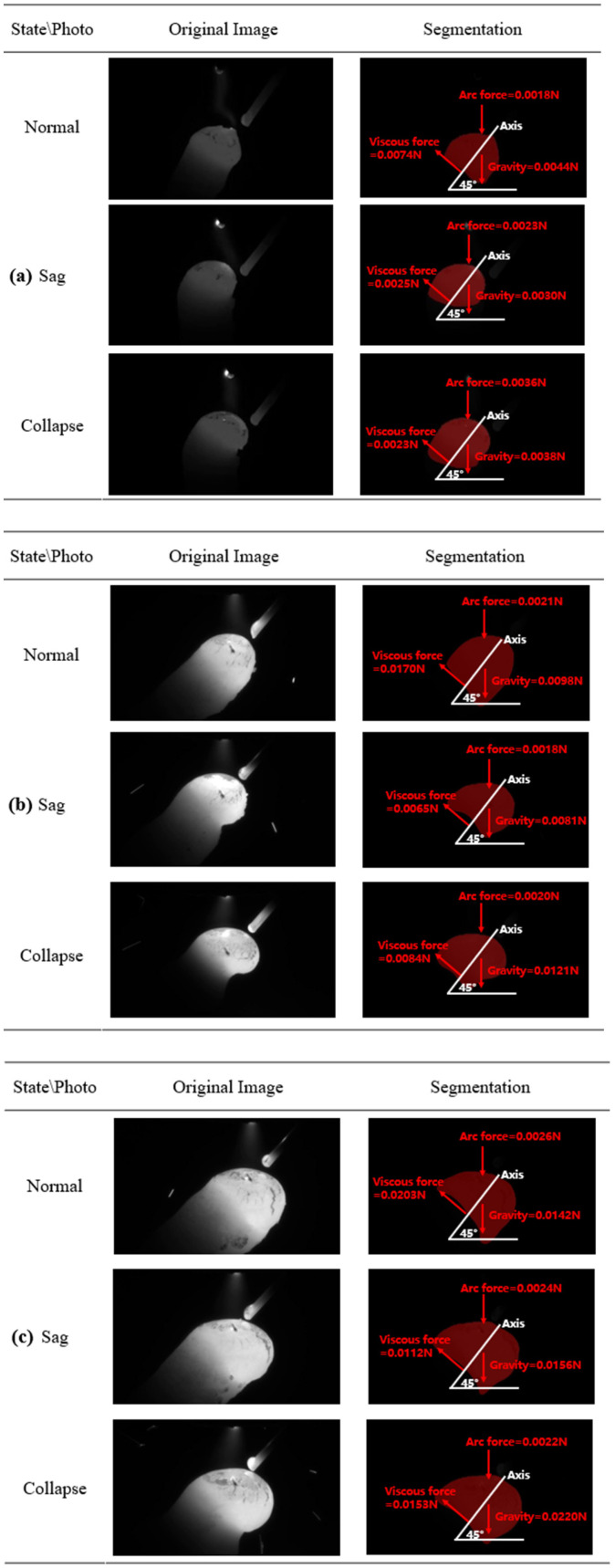
Force analysis results of the melt pool without a support bar: (**a**) The diameter is 6 mm; (**b**) the diameter is 8 mm; and (**c**) the diameter is 10 mm.

Based on the well-designed experimental scheme, we successfully obtained 4 groups of experimental data about unsupported rods with different diameters (6 mm, 8 mm, and 10 mm), totaling 12 groups of data. In order to improve the randomness of the data and test the robustness of the proposed methods, we randomly selected three sets of data from these twelve sets of data, including the base value image and the following peak image. Next, we use the previously proposed method to process each group of experimental data, calculate the specific values of viscous force, gravity, and arc force, and compare them, so as to predict the melt pool state of this group of data. Table 5 lists the calculated results of these experimental data in detail. In addition, Figure 13 depicts the force analysis schematic diagram of the above three groups of melt pool areas of unsupported rods with different diameters: 6 mm, 8 mm, and 10 mm. By examining the numerical results in Table 5 and Figure 13, we can observe that the proposed method effectively predicts the state of the melt pool. Additionally, the proposed methods achieved a high accuracy rate of 90% on the testing set in predicting the melt pool state.

In practice, according to the above steps, we can predict the possible state of the melt pool. Once it is found that the melt pool tends to sag or collapse, we can immediately fine-tune the duty ratio of the control system to allow the melt pool to return to a normal stable state. This measure is of great significance to the whole process of WAAM.

## 5. Discussion

### 5.1. Experimental Evaluation

**Figure 14:**
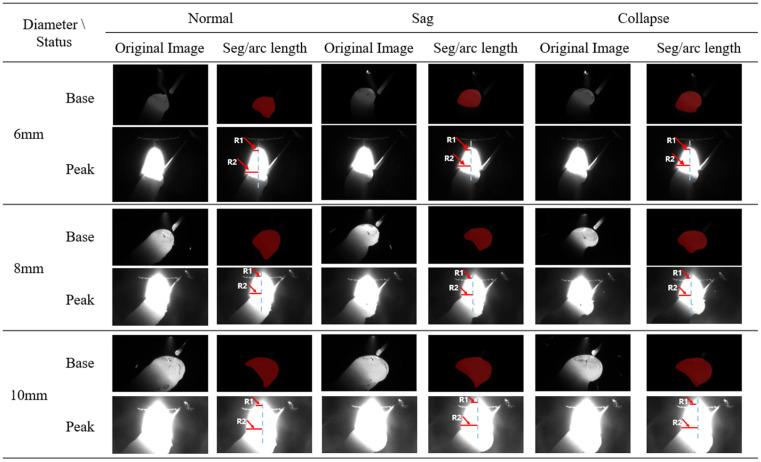
Analysis results of melt pool state of non-support rod with different diameters.

In this experiment, the hardware control system based on visual perception was used to operate on the nearly suspended solidifying melt pool. As a result, we acquired 12 video datasets, each spanning a duration of 2 min. Through data processing and application of the aforementioned vision-based approach, the prediction results for melt pool states (normal, drooping, and collapsing) in cases with 6 mm, 8 mm, and 10 mm diameter non-supported rods were obtained, as shown in Figure 14 for details. As evident from Figure 14, for each rod diameter, it is crucial to extract the melt pool segmentation image and arc length from the base and peak arc images, respectively. By analyzing the segmented image, we find that the proposed segmentation based on improved U-net and ResNet 50 in this paper performed well in the segmentation of the melt pool area. However, an obvious trend is that with the increase in the diameter of the non-supported rod, the effect of melt pool segmentation becomes worse gradually, which is manifested by the partial loss or excessive expansion of the divided area, which inevitably affects the accuracy of the subsequent force balance analysis. We speculate that this is likely due to the unbalanced proportion of images with different melt pool states and acting forces. In addition, the arc radii in the peak state image are analyzed in detail. As can be seen from Figure 14, the arc radius *R*_2_ at the melt pool is easier to measure and the result is more accurate than the arc radius *R*_1_ at the tungsten electrode. This is attributed to the larger size of *R*_2,_ which allows for better distinction of the maximum peak arc radius using computer vision methods. However, the arc radius *R*_1_ at the tungsten tip is very small. Despite this limitation, combined with the results in Table 5 and Figure 13, the proportion of arc force in the force balance analysis of melt pool is not large, so the influence on the overall performance of predicting the melt pool drooping and collapsing states is limited.

In summary, the vision-based force analysis method for the near-suspended melt pool proposed in this study can help better control the state of the melt pool during the printing process for unsupported inclined metal bars in lattice structures. By leveraging the quality control system’s monitoring capabilities, any potential instability in the melt pool, such as sagging or collapse, can be promptly detected. This allows for timely adjustments and control of process parameters, ensuring that each layer of metal can reach a better quality standard and make the printing process stable and efficient.

### 5.2. Result Discussion

In the realm of metal 3D printing technology, the monitoring of the melt pool’s state is crucial for ensuring the quality of metal prints. At present, most of the work on the force analysis of the melt pool remains in theoretical research. Ma et al. [35] delved into the flow behavior of a deformed melt pool under varying lateral gravity conditions and the dynamic evolution of keyholes, elucidating the impact of fluctuations in lateral gravity angles on the fluid dynamics near keyholes. Notably, they observed a significant decrease in the maximum flow velocity of the melt pool as the lateral gravity angle increased. However, this study did not consider methods for monitoring of the melt pool’s state under the influence of lateral gravity. In another perspective, numerous studies have explored the monitoring of the state and morphology of the melt pool using deep learning and vision-based techniques, yet they often fail to analyze the force balance of the melt pool by integrating deep learning algorithms and force analysis theory of melt pool. Cai et al. [27] put forward an image processing method based on U-net to semantically segment the monitoring image during the welding process. Although the results show that this method could accurately extract the contours of the melt pool and keyhole, the force state of the melt pool was not further studied. Xiong et al. [36] used passive vision sensing technology to control the width of the melt pool in gas metal arc additive manufacturing and designed a virtual binocular vision sensing system consisting of a double prism and a camera to monitor the geometric shape of the melt pool. Their experiment showed a detection error of less than 3%, but they refrained from exploring the potential impact of changes in the melt pool width on the force values acting on the melt pool, as well as the risk of subsequent abnormal conditions such as melt pool collapse.

In this study, we integrate theoretical research on the melt pool’s acting force with deep learning and image processing algorithms, introducing an innovative vision-based analysis force balance estimation method for melt pool state prediction. The testing set results indicate a commendable prediction accuracy of 90% for the melt pool state. However, a 10% inaccuracy rate persists, potentially due to the model’s insufficient robustness and the need for an increased number of training samples. In addition, the thermal fluidity of the melt pool at higher temperatures results in slight shrinkage during cooling, posing interference in melt pool area segmentation. Nonetheless, this method offers relatively accurate predictions of the possible states of the melt pool. Consequently, during the actual manufacturing process, prompt human intervention can be initiated upon detecting melt pool instability, and by adjusting the control system’s duty ratio, the melt pool can be stabilized, ultimately enhancing the quality of metal 3D printing.

### 5.3. Limitations

#### 5.3.1. The Diameter of Unsupported Rods

The reason why this study focuses on three kinds of unsupported rods with diameters of 6 mm, 8 mm, and 10 mm for in-depth analysis is that it will be difficult to keep the experimental operation stable when the rod diameter is too small. Although it is theoretically possible to reduce the diameter of the non-supported rod by reducing the arc heat input-per-unit-length, it cannot be reduced indefinitely in the actual manufacturing process. This is because the melt pool is too small to limit the insertion length of wire, which significantly affects the change range of arc length. In addition, the vibration generated by the welding torch during operation can easily make the wire deviate from the melt pool, especially in the initial stage of melt pool deposition, which will further aggravate the instability of arc length and may eventually lead to the failure of the deposition process. Therefore, in order to ensure the stability of the experiment and the reliability of the results, we chose a moderate rod diameter to study.

#### 5.3.2. The Quantity and Quality of Samples

The training effect of deep learning networks depends largely on the quantity and quality of datasets. In this study, there are 600 samples, and the number of samples for normal, drooping, and collapsing melt pools is not balanced, which leads to a drop in performance in dealing with melt pool segmentation for samples with large-diameter rods. Therefore, in order to realize the practical application of this method, we need to further expand the scale of the dataset and ensure that the number of samples in different melt pool states (normal, drooping, and collapse) can be balanced, so as to improve the robustness of the network. In this way, we can train the network more robustly.

## 6. Conclusions

This study is devoted to developing innovative vision-based methods for force balance estimation and abnormal drooping and collapsing melt pool prediction in the process of WAAM. The core of this method is to accurately segment the melt pool using an image semantic segmentation model. Subsequently, with the help of a visual monitoring system and the WAAM equipment, we carried out experiments on three groups of non-supported rods with different diameters and processed the collected experimental data in detail. On this basis, combined with the mechanical formula, we delved into the forces acting on the melt pool, enabling us to accurately forecast its normal, drooping, and collapsing states. The main contributions of this study are as follows:

1. Through experiments and computer vision preprocess technology, the experimental datasets of non-supported rods with 6 mm, 8 mm, and 10 mm diameters were obtained;
2. Based on the unique characteristics of the melt pool imagery, we optimized and adjusted the existing network level of the U-net model. The experimental outcomes demonstrate that the optimized U-net has attained an impressive accuracy of 98.18%, with an MIOU value of 96.64% and an average recall rate of 98.34%;
3. According to the characteristics of the melt pool image, we optimized and adjusted the network design of the U-net model. The experimental results demonstrate that the optimized U-net performed well in the melt pool segmentation task, achieving a high accuracy of 98.18% on the testing set. Additionally, its MIOU value reached 96.64%, and the average recall rate was 98.34%;
4. We successfully obtained key parameters such as the upper contact angle, lower contact angle, top arc length, and bottom arc length from segmented melt pool images via computer vision methods. The forces were then estimated to evaluate the force balance condition, indicating different melt pool states. The results show that the accuracy of our method in predicting the melt pool’s normal, drooping, and collapsing state is as high as 90%.

In summary, the visual perception-based state analysis method for a nearly suspended solidifying pool significantly enhances the automation level and production quality of WAAM. Consequently, the approach introduced in this study serves as an efficient and practical diagnostic tool for identifying abnormalities in melt pools within WAAM, thereby contributing to the advancement of intelligent and unmanned additive manufacturing technology.

## Figures and Tables

**Table 1:** The dimension change in down-sampling feature layers in the proposed melt pool segmentation model.

Module	Characteristic Layer	Dimension
Resize	Layer_1	512 × 512 × 3
	Layer_2	256 × 256 × 64
	Layer_3	128 × 128 × 64
Res_block	Layer_4	128 × 128 × 256
	Layer_5	64 × 64 × 512
	Layer_6	32 × 32 × 1024
	Layer_7	16 × 16 × 2048

**Table 2:** The dimension change in up-sampling feature layers in the proposed melt pool segmentation model.

Module	Characteristic Layer	Dimension
Addition	Layer_8	32 × 32 × 512
	Layer_9	64 × 64 × 256
	Layer_10	128 × 128 × 128
	Layer_11	256 × 256 × 64
Recover	Layer_12	512 × 512 × 64
Segmentation	Layer_13	512 × 512 × 3

**Table 3:** Chemical composition (wt%) of 316 L stainless steel wire.

Element	Fe	C	Cr	Ni	Mo	Mn	Si	Cu	S	P
Wt. (%)	Bal.	0.023	19.00	11.70	2.12	1.85	0.51	0.10	0.013	0.016

**Table 4:** Detailed description of the experimental scheme.

Inclined Angle	Diameter	Parameter Control in the Rod Manufacturing Process
45°	6/8/10 mm	(1) Normal manufacturing in the first 30 s, melt pool drooping in 30–60 s, normal manufacturing in 60–90 s, and melt pool collapsing in 90–120 s.
		(2) Normal manufacturing in the first 30 s, melt pool collapsing in 30–60 s, normal manufacturing in 60–90 s, and melt pool drooping in 90–120 s.
		(3) Normal manufacturing in the first 45 s, melt pool drooping in 45–75 s, normal manufacturing in 75–90 s, and melt pool collapsing in 90–120 s.
		(4) Normal manufacturing in the first 45 s, melt pool collapsing in 45–75 s, normal manufacturing in 75–90 s, and melt pool drooping in 90–120 s.

**Table 5:** Force balance estimation results of the melt pool in different states.

Diameter\Status (Unit: 10^−3^ N)	Normal			Sag			Collapse		
	Viscous Force	Gravity	Arc Force	Viscous Force	Gravity	Arc Force	Viscous Force	Gravity	Arc Force
6 mm	7.4	4.4	1.8	2.5	3.0	2.3	2.3	3.8	3.6
8 mm	17.0	9.8	2.1	6.5	8.1	1.8	8.4	12.1	2.0
10 mm	20.3	14.2	2.6	11.2	15.6	2.4	15.3	22.0	2.2

## Data Availability

The data presented in this study are available on request from the corresponding author.
